# Assessing the implementation and effectiveness of early integrated palliative care in long-term care facilities in France: an interventional mixed-methods study protocol

**DOI:** 10.1186/s12904-023-01157-w

**Published:** 2023-04-06

**Authors:** Emmanuel Bagaragaza, Isabelle Colombet, Mireille Perineau, Philippe Aegerter, Frédéric Guirimand

**Affiliations:** 1 Maison Médicale Jeanne Garnier, Département Recherche Enseignement Formation (DREF), 106 avenue Emile Zola 106-108 Avenue Emile Zola, Paris, 75015 France; 2 Université Paris Cité, Paris, France; 3 Centre Hospitalier d’Avignon, 305A Rue Raoul Follereau, Avignon, 84000 France; 4 Université de Versailles Saint-Quentin-en-Yvelines Département Santé Publique - U1018 UVSQ INSERM, GIRCI IdF, 2 Av. de la Source de la Bièvre, Montigny-le-Bretonneux, 78180 France

**Keywords:** Older persons, Early palliative care, Interventional research, Long-term care, Implementation, Quality improvement, Stepped wedge cluster randomised trial

## Abstract

**Background:**

Majority of residents in long-term care facilities (LTCF) have limited and delayed access to palliative care even though many suffer from incurable chronic illnesses that will likely require the provision of palliative care. We present the study protocol of “PADI-Palli”, an intervention aims to advance early integrated palliative care into standard care delivered in LTCF. This study will assess the effectiveness of early integrated palliative care on palliative care accessibility for older persons in LTCF, and identify the key factors for the successful implementation of early integrated palliative care and its sustainability in the LTCF context.

**Methods:**

This multicentre interventional study utilises a pragmatic research design with a convergent parallel mixed-methods approach. The qualitative study will use a case study design and the quantitative study will use a stepped wedge cluster randomised trial. In total, 21 participating LTCF from three French regions will be randomly allocated to one of seven clusters. The clusters will cross over from the usual care to the active intervention condition over the course of the study. The primary outcome relates to the accurate identification of palliative care needs and early access to palliative care for LTCF residents. Secondary outcomes are quality of care, quality of life for residents and their families, and quality of life at work for professionals. Measurements will be performed before and after the intervention. Implementation and evaluation of PADI-Palli intervention is grounded in the Consolidated Framework for Implementation Research.

**Discussion:**

Existing evidence demonstrates that early integrated palliative care in cancer care leads to a significant improvement in patient outcomes and processes of care. Little is known, however, about early integrated palliative care in the context of LTCF for older persons. This study has the potential to fill this gap in the literature by providing evidence on the effectiveness of early integrated palliative care for older persons in LTCF. Moreover, this study will provide a better understanding of the relevant contextual elements that facilitate or hinder early integrated palliative care implementation and transferability. If proven effective, this intervention can be scaled to other care settings in which older persons require palliative care.

**Trial registration:**

ClinicalTrials.gov ID: NCT04708002; National registration: ID-RCB number: 2020-A01832-37.

## Background

The World Health Organization (WHO) and national health authorities such as the French National Authority for Health (HAS) recommend the implementation of an early integrated palliative approach in the care pathway of all patients, including older persons, afflicted by incurable illness [1, 2]. Long-term care facilities (LTCF), known as EHPAD (Établissement d’Hébergement pour Personnes Agées Dépendantes) in France, are admitting a growing number of elderly residents afflicted by advanced chronic pathologies [3], and the majority of these individuals require palliative care services [4]. In France, the number of LTCF residents who die each year represents one quarter of the total annual deaths of all ages [5]. However, despite the number of residents with multiple serious chronic conditions and the high mortality rates in long-term care settings, the provision of palliative care to LTCF residents remains limited and delayed to the last weeks, or even days, before death [6], and palliative care approach is rarely discussed with residents or their families [7].

Non-specialist health professionals working in palliative care are often unaware of the benefits of palliative care for both the patient and their family [8], which results in skills gaps in pain management, advanced care planning, and end-of-life care [9]. Accordingly, the use of specialised palliative care services are introduced late in the trajectory of care, with a median of three to four weeks before death, and even later in geriatric centres, with a median of seven days before death [10]. Moreover, palliative care delivery is often driven by the patient’s diagnosis and subsequent prognosis rather than by the current needs of the patient [11]. Consequently, the deficit of palliative care provision in LTCF leads to the excessive and inappropriate use of acute health services, including emergency visits and unscheduled hospitalizations [12]. Current evidence points to a significant burden of unrelieved symptoms among residents at the end of life [13], and a lack of effective symptom assessment and management [14]. In addition, person-centred care is not adopted and the expectations of relatives are not satisfied, which negatively affects the quality of life of LTCF residents and their families [15]. Finally, care providers in LTCF settings confronted with death situations and complex care needs beyond their competencies are susceptible to psychological stress and a diminished quality of life at work [16].

The WHO defines the palliative care approach as designed to provide person-centred care, respecting personal preferences and human dignity [17]. The palliative care approach focuses on preventing and relieving all dimensions of suffering and maintaining quality of life in the context of incurable and complex pathology [18]. In cancer care, early integrated palliative care allows a better anticipation of the patient’s preference for end-of-life care and a more effective prevention of physical or psychological suffering, therefore increasing quality of life without decreasing survival [19–23]. A study by Nieder and colleagues shows that people who received early integrated palliative care are less often hospitalised in the last three months of life (73% vs. 97%), die less often in the hospital (33% vs. 48%), and have their wishes documented earlier and more thoroughly [24]. According to Hui and Bruera, integrating palliative care early in the care pathway improves symptom control, quality of end-of-life care, caregiver satisfaction, and patient quality of life and satisfaction [25]. However, the provision of palliative care decreases in the absence of cancer or with ageing [26]. In fact, older persons with neurodegenerative diseases rarely benefit from palliative care and research specific to palliative care services for this population is scarce [27].

Despite available evidence on the benefits of palliative care, and various models of early integration of palliative care within the cancer care context, its effectiveness and transferability within the LTCF context for older persons is poorly documented. Furthermore, studies rarely apply an implementation science lens to enhance implementation processes and improve the effectiveness of early palliative care integrated into existing care practices. Thus, only a handful of studies have analysed contextual factors that might mediate the successful implementation of an early integrated palliative care approach within LTCF [28].

To assist in filling this gap, we designed an interventional research model named “PADI-Palli” (Personne Agée Demarche PALLIative Intégrée). Its aims are to advance early integrated palliative care in LTCF to improve palliative care access, prevent and relieve the suffering of older persons, support the best possible quality of life for residents and their families, and improve the quality of life at work for LTCF professionals.

In this study, the following research questions will be addressed: (1) To what extent was the early integrated palliative care model implemented as intended? (2) What were the factors that facilitated and hindered the implementation of early integrated palliative care in LTCF? (3) Does early integrated palliative care contribute to improved timely access to palliative care, quality of care, quality of life of residents, and quality of life at work for LTCF professionals?

Thus, the primary objective of this study is to assess the effectiveness of early integrated palliative care implementation in LTCF in terms of timely access to palliative care for older residents. This study also has two secondary objectives: (1) To identify factors that facilitate or hinder the implementation of early integrated palliative care and its sustainability in the LTCF context, and (2) To assess the effectiveness of early integrated palliative care in LTCF on (a) quality of palliative care services delivered (b) care pathway, (c) quality of life of residents and that of their family caregivers, and (d) quality of life at work for LTCF professionals.

We postulated that early integrated palliative care implementation should support person-centred care practices [29] and strengthen integrated care pathway for residents [27], and as a result improve quality of care and quality of life for LTCF residents and their relatives as well as quality of life at work for LTCF professionals. Therefore, the primary hypothesis addressed in this study is that implementing the early integrated palliative care should improve the timely identification of palliative care needs and access to palliative care for residents in LTCF with serious advanced chronic conditions requiring palliative care provision. The secondary hypotheses are: (1) Implementing early integrated palliative care should improve the quality of care delivered to LTCF residents; (2) Implementing early integrated palliative care should improve the quality of life of residents and their caregivers; and (3) Implementing early integrated palliative care should improve the quality of life at work for LTCF professionals.

## Methods

### Intervention

#### Developing early integrated palliative care tailored to LTCF settings

Addressing the problems related to the challenging nature of palliative situations necessitates an intervention model and implementation methods tailored to their complexity [30]. Therefore, involving all stakeholders in the development of suitable interventions and using a theory-based evidence-driven approach are recommended [31–33]. To develop an appropriate intervention for early integrated palliative care in LTCF, we referenced the Medical Research Council (MRC) framework, which uses four different phases to develop and evaluate complex interventions: development (0-I), feasibility (II), evaluation (III), and implementation (dissemination and long-term follow-up) (IV) [31].

The phases 0 and I started with the identification of existing evidence-based literature for early and integrated palliative care to inform the core of PADI-Palli intervention. The research team (EB, MP, PA, & FG) developed the theoretical components of the intervention and constructed a draft model of early integrated palliative care. We conducted a pilot study from 2017 to 2018 in order to: (1) understand the challenges associated with palliative care in LTCF settings, and (2) test a theoretical model of palliative care implementation appropriate to this context. The research team met with healthcare professionals and managers of 10 LTCF and experts in palliative care to gain an understanding of the situation and needs in LTCF settings to be included as the relevant components of the intervention. These informed the development of the PADI-Palli intervention model with six key components (Table 1). In line with the MRC recommendations, the feasibility of delivering the key elements of the intervention and their acceptability were tested in 10 LTCF (MRC- phase II).

The study protocol reported in this paper is categorized as MRC- phase III, which corresponds to the empirical evaluation of early integrated palliative care, implementation analysis, and effectiveness evaluation in real-world organization of LTCF settings.

**Table 1:** Key components and expected outcomes of PADI-Palli intervention model

Key components	Expected outcomes
1: Shared vision of palliative care approach: sharing of experience and existing knowledge, co-creation and application of new knowledge	- Shared views and understanding of palliative care. - Changed previously held (mis)beliefs and acquisition of new knowledge related to palliative care and early integrated palliative care. - Adoption of evidence-based palliative practices.
2: Interprofessional companionship “Compagnonnage”	- Strengthened collaboration within LTCF. - Improved palliative care practices at individual and collective levels. - Improved quality of life at work for healthcare professionals.
3: Use of validated tools for clinical assessment (identification and patient needs assessment)	- Improved knowledge and utilization of validated tools for the assessment of palliative care needs in older persons.
4: Early pro-active identification	- Improved knowledge and utilization of validated tools for the pro-active identification of residents who may benefit from early palliative care.
5: Advance care planning	- Change of misconception of residents and their caregivers towards palliative care. - Improved quality of care. - Improved quality of life for older persons and their caregivers.
6: Interprofessional and intersectoral collaboration between health and medical-social fields/services	- Enhanced partnership between LTCF, hospitals, and specialist palliative care teams. - Improved quality of life at work and reduced distress for professionals in relation to end-of-life care management.

#### Implementation strategy

As recommended in the implementation sciences, we adopted an implementation framework for enhancing implementation effectiveness [34] and for understanding the conditions necessary to support the introduction of PADI-Palli intervention, all the while meeting specific local situation and setting requirements [35]. Thus, implementation and evaluation of PADI-Palli intervention are grounded in the Consolidated Framework for Implementation Research (CFIR) [36]. The CIFR is regarded as a helpful theoretical framework for implementation research on healthcare service delivery as it allows for the consideration of specific determinants that can influence implementation outcomes and the assessment of implementation effectiveness under five domains: intervention characteristics, outer setting, inner setting, characteristics of individuals, and process [34]. Given that everyday micro-level components of clinical practice, organizational dynamics within LTCF, and external factors can potentially influence the effectiveness of the palliative care approach, these factors should be considered as a whole system [35].

To reach the expected change goals at these different levels and facilitate the implementation, analysis, and transferability of PADI-Palli intervention, our implementation strategy is informed by Rogers’ theory of innovation decision process [37]. According to this theory, the characteristics of implementation settings at individual and system levels, the existing knowledge and practices, and the stakeholders’ expectations should be considered throughout the decision-making process and intervention implementation.

The implementation is categorized into three periods with six overall steps. Period one (pre-implementation) includes two steps, identification and engagement of participants and evaluation of the current state in terms of palliative care practices, professional competencies, palliative care needs, and organisational characteristics; period two (implementation) has two steps, intervention delivery and consolidation; and period three (post-implementation) with two steps, autonomy and sustainability.

### Process evaluation design: a parallel mixed-methods approach

**Fig. 1:**
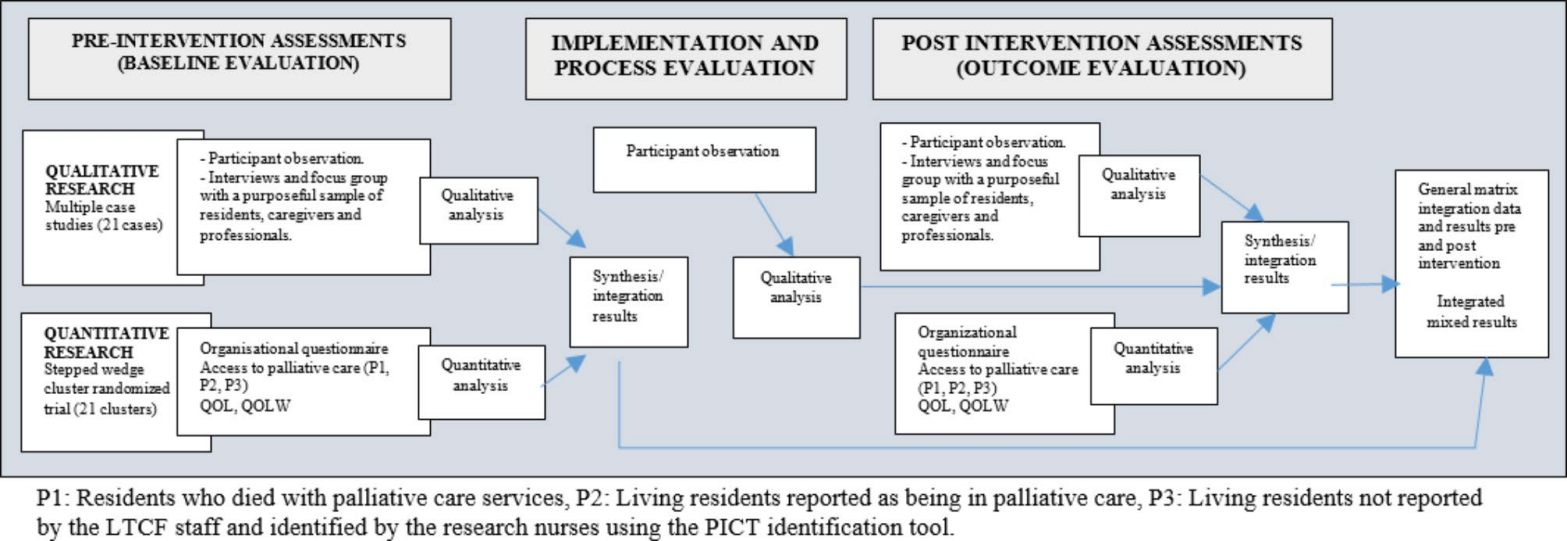
Flowchart representing the research design with a convergent parallel mixed-method

This study is a multicentre interventional research using a pragmatic study design [38] with a convergent parallel mixed-method approach [39] (Fig. 1). The qualitative component of the study follows a case study methodology [40], and the quantitative component of the study involves a stepped wedge cluster randomised trial (SW-CRT) [41].

The clusters will cross over from the usual to the active intervention condition during the trial, according to a randomised schedule. This design is adapted to collect intervention data and is used to evaluate an intervention during routine implementation [42]. While cluster randomisation addresses the problem of contamination bias, staggering the intervention among clusters controls for temporal drift and facilitates the logistics of implementation and data collection [43]. The principle that all study sites will benefit from the intervention is an important motivator that contributes to the feasibility of this study.

**Fig. 2:**
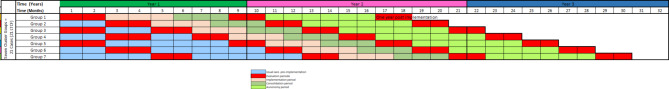
Stepped wedge with seven cluster groups and periods of measurements. The clusters were randomly assigned to seven groups (One Cluster = 1 LTCF) that crossover to receive the intervention after pre-intervention measurements (one to two measurements).

As depicted below in Fig. 2, over the course of the study timeline each of the 21 participating centres will be defined as a cluster and randomly allocated to one of the seven groups. This allocation will follow a sequence similar to the observational and interventional periods. Randomisation of LTCF will be stratified by region; each group includes LTCF from the three participating regions.

The strategy used addresses issues related to incomplete design by minimizing the collection load to preserve good power by removing uninformative combinations of clusters and periods without too much alteration of the power [44]. A sequence includes: one or two pre-intervention observational periods of two months; one delivery intervention period of three months followed by an interventional consolidation period of three months; then a short-term observational period of two months; and 12 months after intervention a final observational period of two months, called autonomy.

The individual data collection is based on an open cohort: as the study progresses, some residents will leave the LTCF or die (on average 25% each year, but likely higher for subjects requiring palliative care), while others will be admitted to the LTCF or begin to require palliative care and will be included at a later date.

### Outcome evaluation

#### Primary outcome measures

The primary outcome measures relate to the accurate identification of palliative care needs and early access to palliative care for residents in LTCF context. These will be assessed through a comparison of the pre- and post-intervention periods, with the main comparison focusing on the latest evaluation (autonomy). The following will be measured and compared:

1) Identification sensitivity: This main criterion is the proportion of residents identified by the LTCF team as in need of palliative care among those with “potential” palliative care needs identified proactively by a dedicated research nurse using “Gold Standards Framework Proactive Identification Guidance” [45] and the Palliative Care Indicator Tool (PICT) [46].

2) Access: The proportion of residents with effective access to palliative care delivery among those identified as in need of palliative care (with or without referral to the specialist palliative care service).

3) Optimal timing: We postulate that during the pre-intervention phase, the identification of residents’ palliative care needs is delayed and may not be systematically followed by the delivery of palliative care. Therefore, we will estimate the time gain by focusing on the interval between the time residents are identified as in need of palliative care and the time of their death (or discharge, withdraw from follow-up). An increase in this time can be interpreted as a shortening of the identification delay but also as an extension of the delivery period of palliative care and the identification of the need for palliative care, both of which would support the effectiveness of palliative care.

#### Secondary outcome measures

The secondary outcomes relate to the effectiveness of early integrated palliative care on (1) quality of care and healthcare services utilisation, (2) quality of life for residents and their families, and (3) quality of life at work for LTCF professionals. These expected outcomes are connected to the overall goal of palliative care, which is to improve quality of life for individuals and their families [17], and the quality perceived by relatives can influence patients’ quality of life [47]. In addition, early integrated palliative care can have a potential effect on the care pathway [48] and on the working conditions of healthcare providers [49] which could affect their quality of life [50, 51].

##### Quality of care

will be assessed in two ways: (i) by the palliative care quality indicators extracted from InterRAI-Palliative Care (InterRAI-PC) instrument [52] and (ii) by perceived quality of care as expressed by relatives of residents using the QUALI PALLI FAM questionnaire [47]. Several domains will be measured including symptom management (prevalence of negative mood, prevalence of pain that is not controlled), caregiver support (prevalence of caregiver’s distress), information and communication, coordination of care, and spiritual and emotional care support. Both tools have adequate reliability and validity [47, 52].

##### Quality of life (QoL)

for residents will be measured using the interRAI Self-Reported Quality of Life (interRAI-QOL) [53]. The InterRAI-QOL measures 10 domains: privacy, food/meal, safety/security, comfort, autonomy, respect, responsiveness of staff, staff-resident bonding, activity options, personal relationships (presence of friends). The minimum score (0) is the best outcome for quality of life, and the maximum (16–24) is the worst outcome.

##### Quality of life of informal caregivers

will be assessed using the Caregiver’s Burden Scale in End-of-Life Care (CBS-EOLC) self-questionnaire [54]. A minimum score of 16 indicates the best quality of life for informal caregivers, whereas a maximum score of 54 indicates the worst quality of life.

##### Professional quality of life at work

will be assessed using the 30-item Professional Quality of Life (ProQoL R-V) scale that measures compassion fatigue, compassion satisfaction, and burnout [55, 56]. Compassion satisfaction is defined as the pleasure derived from being able to do the work well while compassion fatigue results from giving high levels of energy and compassion over a prolonged period to those who are suffering, often without experiencing the positive outcomes of seeing patients improve [57], which seems particularly relevant in the LTCF context. The highest score suggests a good quality of life of professionals and the lowest score suggests a poor quality of life.

### Settings and participants

#### Settings

The settings of this interventional study includes 21 LTCF in three different regions of France (Ile de France, Provence-Alpes-Côte d’Azur, and Nouvelle-Aquitaine). The LTCF were recruited based on a group of seven LTCF settings per region. Purposeful sampling strategy was used for the LTCF selection. Attention was given to obtain a mix of characteristics: funding (public, private for-profit or not), geographical location (urban/non-urban territory), size (bed capacity), and affiliation or not with a hospital.

#### Participants and eligibility criteria

The study targets three categories of population: LTCF residents in need of palliative care, their family caregivers, and healthcare providers.

Recruitment of residents will be done continuously over time. All older persons aged 60 years and above who are residents in one of the participating LTCF will be potentially eligible for screening for palliative care needs. If a LTCF is too large to be included entirely, a part of the site (building, wing, floor) will be randomly selected.

For each resident identified with palliative care needs, their main informal caregiver will also be identified. Informal caregivers could be any person considered by the resident or the professional caregivers as an informal caregiver, regardless of whether this individual is a family member of the resident. With the resident’s consent, their informal caregiver will be invited to participate.

All healthcare providers and managers from the participating sites are eligible for the study, except those who will voluntarily decline to participate and those who are on a temporary work contract at the LTCF.

#### Sample size and power calculations

Proper identification of palliative care needs for a resident, the main primary outcome, served as the basis for calculation of the number of subjects required for the quantitative component of the study. Power and sample size calculations were performed using simulations written in R language based on the approach described by Baio and colleagues, and Hemming and colleagues [58, 59].

The following parameters were used in the program: number of LTCF, number of waves (i.e. groups of LTCF) from three to seven, proportion of eligible residents in need of palliative care (from 20 to 40%, size of an average LTCF is 100 residents), and proportion of identification within that sub-population (from 20 to 50%). We also considered the expected absolute minimum increase in identification of 15–30%, intra-cluster correlation (from 0.02 to 0.1), intra-individual correlation from zero (cross-sectional design) to 0.5 (cohort design) according to the open cohort design, and the fact that a resident may participate in one (cross-sectional design) to four (closed-cohort design) periods and provide as many observations. To highlight an absolute increase of 15 points to 20 points in the proportion of the residents needing palliative care and having been identified, with a 5% alpha risk, 80% power, the number of observations needed to range from 1000 to 2000. As an average LTCF may provide 25 eligible residents which may be observed two times, our 21 LTCF set has the potential for 1000 observations. Elsewhere, an increase in detection of palliative care from 50 to 65%, or from 40 to 60%, corresponds to an effect size of 0.3 or 0.4, respectively. By comparison, the Quebec trial of a multifaceted intervention on the quality of the end of life of dementia patients in long-term care showed effect sizes of 0.4 for the CAD-EOLD score measuring comfort during dying and 0.34 for peaceful death [60].

For the qualitative component, we will use a nonprobability purposive sampling to include a range of participants who could inform a deeper understanding of the phenomena investigated.

### Data collection procedures

As this study uses a convergent parallel mixed-method design [39], quantitative and qualitative data will be collected at the same time from each cluster considered a case. In accordance with the stepped wedge design, the measurements will be carried out simultaneously in all sites of the same group during the pre-intervention period (one to two measurements depending on the cluster), once at the end of the intervention period and once at the 12th month post-intervention (Fig. 2). There will be no quantitative data collected during the intervention delivery period.

Resident quantitative data will be retrieved using PICT and a subset of InterRAI-PC scales. Dedicated nurses with experience in palliative care will serve as research assistants after receiving training on the use of the above tools and the multidimensional evaluation approach. They will register data in the RAisoft database.

To collect descriptive and explanatory variables at the LTCF level, our research team developed an organizational questionnaire with two parts: (i) LTCF characteristics and functionalities: size and skill set of human resources, coordination with other community and hospital services in palliative care; and (ii) sociodemographic and medical characteristics, overall death rate of residents at the entire site.

To complement the tools used to measure outcomes, the research team developed a structured checklist of indicators to assess retrospectively and prospectively the accessibility and quality of care provided to residents with palliative care needs. Trained research assistants will retrospectively review the files of the residents for six months before the pre-implementation period and throughout the study, as well as prospectively follow-up with prospective residents with palliative care needs. Items related to consideration of residents’ needs and preferences, symptom management, resident care pathway and health service use will be checked.

Qualitative data will be collected continuously throughout the study duration. We will use a triangulation approach to data collection: individual interviews, focus groups, and participant observations, from multiple data sources.

Individual in-depth semi-structured interviews will be conducted during the pre- and post-implementation phases with professionals and managers, informal caregivers, and residents from each site, as well as with specialised palliative support teams affiliated with the participating LTCF. In addition, focus group discussions with various healthcare professionals involved in the direct care of residents (nurses, nurse assistants, psychologists, physiotherapists, occupational therapists) from nine purposively selected cases will be conducted. Interview guides specific to each group of participants will serve as data collection tools. Four themes will be explored throughout the interviews and focus groups: current practices, palliative care perceptions, quality of life at work, and barriers and facilitators of the implementation of early integrated palliative care. A researcher trained in qualitative research will conduct the individual interviews and focus groups.

Participant observations will be carried out throughout different periods of study. An observation guide was developed to collect information during the observation of care situations in LTCF. Information will be collected on several items at each LTCF, including usual palliative care practices, level of quality of routine service, processes, structural characteristics, organizational culture and professional interactions, and external factors such as the exiting of support teams in palliative care services provision and the territorial provision/organisation of palliative care. Extensive field notes will be taken, and a weekly research team meeting will be held to continuously analyse the collected observations.

### Analysis

#### Quantitative analysis

The quantitative data will be analysed using R. Statistical analysis and will be performed after a blind review of data quality issues and a database “freeze.” Due to the sequence of interventions, the analysis cannot be carried out in a blinded fashion.

A patient flow chart will be set up according to the CONSORT extension recommendations for stepped wedge design [61]. Main analysis will be performed on all included residents following the intent-to-treat principle and according to the role assigned to each period (usual care in the pre-intervention period versus experimental care in the post-intervention period) regardless of whether palliative care was implemented.

#### Descriptive analysis

The descriptive analysis will summarize baseline clinical characteristics of the study cohort. Descriptive statistics will be reported as means and medians, standard deviations, and interquartile ranges. The nominal data will be reported as percentages with two-sided 95% confidence intervals. The proportions of missing data will be calculated. Participant characteristics (eligibility criteria, demographics data at inclusion) will be described and compared by setting and reported without comparison by period.

#### Primary outcome analysis

The data analysis will be based on the comparison of data collected during pre-intervention versus post-intervention periods. The three primary criteria will be tested according to a hierarchical procedure: identification sensitivity, access, and optimal timing from a fixed sequence procedure; constructed using a pre-specified sequence of hypotheses; and endpoints ordered according to their importance [62]. All tests will be performed at the 0.05 level following the pre-specified order. Once one hypothesis is tested and the results indicate it is not significant, all subsequent tests will not be performed.

The statistical model will take into account the non-independence of the observations, as multiple evaluations may concern the same resident across time due to the use of open cohorts and several residents are included from the same LTCF. This hierarchy (three levels) leads to the use of generalized linear mixed models (GLMM) for repeated measures [63]. For dichotomous outcomes (i.e. the patient has been identified or not), we will use a Bernoulli distribution with a logistic link function, and for continuous outcomes a normal distribution with identity link function. The independent variables will be: the LTCF and the resident considered as random factors, the calendar time interval (common to all groups) considered as a fixed factor, and treatment mode for the cluster in the time interval (pre-intervention = control, post-intervention = experimental) considered as a fixed factor associated with the effect of the intervention. The chronological sequence of stepped wedge randomized trials means that the number of clusters exposed to the intervention being tested increases as the trial progresses [41].

The observation of an intervention effect may reflect the existence of an underlying secular trend that is independent of the intervention. Therefore, modelling the background secular trend is necessary to remove bias in estimating the effect attributed solely to the intervention. However, the saturated time parameterization (by J-1 indicators if there are J periods) may not be the most efficient approach if there are a large number of periods relative to the number of waves and clusters due to the reduced degree of freedom available for estimating the intervention effect. Thus, we will use a parsimonious specification with a minimum number of parameters, such as a polynomial specification to a fixed degree [64].

To test the consistency of the hypothesis of a constant effect or conversely a time-varying intervention effect, we will develop a similar model with a random effects covariate representing the interaction of time by cluster. Finally, there may be variation across clusters in the magnitude of intervention effects that will be modelled by a cluster-period-specific random effect deviation [65] as this type of modelling also takes into account the incomplete structure of the stepped wedge design.

As additional exploratory analysis, the individual and institutional characteristics as well as the intensity of intervention will be used as explanatory or confounder factors as recommended in the literature and by expert opinion. In order to minimize the dimensionality of these factors, principal component analysis or a Lasso-type technique could be applied. The possible nonlinear effects of the continuous variables will be managed through fractional polynomials. The models’ performance will be assessed by the Akaike information criterion [66], and the stability of the estimates will be checked by resampling.

#### Secondary outcome analysis

Multilevel multivariate models will be constructed and validated as in the analysis of the primary outcome analysis. The survival time of residents will be analysed by a Cox model including a delayed input, a temporal axis representing age of admission in LTCF as its origin, and the situation with respect to the intervention coded by a time-dependant variable.

### Qualitative analysis

A thematic content analysis [67] approach will guide the qualitative analysis of data, and continuous ascending coding will be privileged. The analysis will focus on both latent and manifest content. A team of three researchers with expertise in qualitative research will simultaneously conduct the qualitative analysis. To ensure a triangulated analysis approach, each of the three researchers will identify themes, build and validate the thematic tree. The unit of analysis will be the organisational level of each LTCF, considered as a case. NVivo software version 12 will facilitate qualitative data management, storage, and coding.

Pre- and post-implementation analysis will combine both interviews and observation data. The CFIR framework will guide the analysis of factors that shaped the implementation and sustainability of early integrated palliative care intervention.

### Integrative analysis

The qualitative and quantitative analyses will be completed with an integrative analysis that uses intra- and inter-cases to identify similarities and differences. This approach will enrich the understanding of any internal and external patterns and variances identified in each case.

The integration of qualitative and quantitative data will be carried out at each stage of analysis to perform an integrative analysis and interpretation. The results from the qualitative study will be triangulated with the results from the quantitative study, according to the research question being addressed, and will determine the integrative framework and different types of data that will be used and brought together in the analysis.

### Ethics approval and informed consent

Ethics approval for the conduct of this study was granted by the French Committee of Protection of Person (CPP), approval number 2020.09.06 bis 20.07.31.64318.

All participants (LTCF residents, family caregivers, and professionals) will be required to provide their informed consent in writing before their inclusion in the study. For residents who are unable to provide informed consent, this will be obtained from their legal representatives. Participant confidentiality will be ensured using codes and pseudonyms. De-identified data will be stored in protected computers and dedicated locked space. The use of databases and data processing are implemented in accordance with European regulations (General Data Protection Regulation - GDPR dated April 27, 2016).

## Discussion

Early integration of palliative care into the care pathway of older people is a challenging public health issue. To improve access to palliative care for people who are in need of such care, the integration of palliative care into the overall healthcare system is recommended [11]. This is a multicentre interventional study that aims to assess the effectiveness of early integrated palliative care on palliative care accessibility for older persons in LTCF and to identify the key factors for the successful implementation of early integrated palliative care and its sustainability in the LTCF context.

Previous studies have substantially demonstrated the benefits of early palliative care for patients with cancer [19, 20] and for their informal caregivers [68]. A wide range of strategies for integrating palliative care are recommended and shown to be effective [22, 23]. However, a pilot study conducted before designing the current project confirmed what is widely documented in the literature—there is an insufficient use of palliative care for the elderly, particularly for those with cognitive impairments [7] or with an absence of cancerous pathologies [26]. Evidence on the effectiveness of an early integrated palliative care approach for the elderly in LTCF is yet to be developed in France.

While factors limiting the development and implementation of palliative care are documented [69, 70], strategies for the integration of early palliative care in real-world LTCF settings were often overlooked. The mediating factors of the early integration of palliative care and its sustainability in LTCF contexts are poorly documented, if documented at all, in the French context. This study intends to provide relevant information on the effective integration of early palliative care in LTCF settings, as well as updated evidence on factors that influence the implementation process of early palliative care and its transferability across LTCF settings.

The development of the PADI-Palli intervention and its implementation utilises evidence-based guideline recommendations and theoretical frameworks from implementation sciences, and considers the practical issues experienced by those in LTCF by engaging key stakeholders. The use of this theory-based evidence-driven approach should increase both intervention effectiveness and transferability [33]. In addition, as recommended in interventional studies [71], our study uses a mixed-methods approach to overcome the challenges typically faced in these types of studies and interventional evaluations. The stepped wedge implementation design enables comparisons within (pre- and post-intervention) and between clusters (inter-sites). It maximises statistical power and allows the testing of whether an intervention is effective in real-world settings. The qualitative approach will enable a better understanding of relevant contextual factors that facilitate or hinder early integrated palliative care implementation, and it will underpin the reflection at the time of the implementation and assist with the consolidation of the intervention.

Although using the stepped wedge design in combination with a qualitative study can be logistically challenging, there are several advantages to this approach. Not only does this design include methodological and ethical considerations, but also it facilitates a better understanding of the study results, including changes or lack thereof, and the factors that contributed the results. This study is conducted in accordance with the trustworthiness and rigour criteria applied to the quantitative approach: internal validity, external validity, reliability, and objectivity [39]; and the qualitative approach: dependability, confirmability, credibility and transferability [72]. The publication of this paper describing the methodology and conduct of this study contributes to these validity criteria, including transferability.

The richness of the results from this multicentre interventional study has the potential to allow a better exploration and understanding of the contextual influences shaping intervention activities and outcomes of early integrated palliative care within LTFC. If proven effective, this intervention can be scaled to other care settings in which older persons require palliative care. The results will be disseminated through presentations at scientific conferences and professional meetings and publication in peer-reviewed scientific journals.

## Data Availability

Not applicable.
